# The influence of the precuneus on the medial temporal cortex determines the subjective quality of memory during the retrieval of naturalistic episodes

**DOI:** 10.1038/s41598-024-58298-y

**Published:** 2024-04-04

**Authors:** Samy-Adrien Foudil, Emiliano Macaluso

**Affiliations:** 1 Université Claude Bernard Lyon 1, CNRS, INSERM, Centre de Recherche en Neurosciences de Lyon (CRNL), U1028 UMR5292, IMPACT, 69500 Bron, France; 2 Lyon Neuroscience Research Center (ImpAct Team), 16 Avenue Doyen Lépine, 69500 Bron, France

**Keywords:** Long-term memory, Cognitive neuroscience

## Abstract

Memory retrieval entails dynamic interactions between the medial temporal lobe and areas in the parietal and frontal cortices. Here, we tested the hypothesis that effective connectivity between the precuneus, in the medial parietal cortex, and the medial temporal cortex contributes to the subjective quality of remembering objects together with information about their rich spatio-temporal encoding context. During a 45 min encoding session, the participants were presented with pictures of objects while they actively explored a virtual town. The following day, under fMRI, participants were presented with images of objects and had to report whether: they recognized the object and could remember the place/time of encoding, the object was familiar only, or the object was new. The hippocampus/parahippocampus, the precuneus and the ventro-medial prefrontal cortex activated when the participants successfully recognized objects they had seen in the virtual town and reported that they could remember the place/time of these events. Analyses of effective connectivity showed that the influence exerted by the precuneus on the medial temporal cortex mediates this effect of episodic recollection. Our findings demonstrate the role of the inter-regional connectivity in mediating the subjective experience of remembering and underline the relevance of studying memory in contextually-rich conditions.

## Introduction

The neural basis of episodic memory has been extensively investigated in both humans and animal models. Traditional views posit a central role of the what-where-when of episodic events, engaging the medial temporal lobe (MTL)^1,2^ and the prefrontal cortex^3,4^. The parietal cortex has also been found to contribute to episodic memory^5,6^. Areas in the lateral parietal cortex have been associated with the quality of the retrieval, such as the richness and the confidence of the retrieved information^7,8^. The medial parietal cortex has been associated with memory for contextual details^9–11^, memory confidence^12^ and vividness^13^, emphasizing retrieval aspects related to the subjective experience of recollection. Medial parietal regions, including the precuneus, participate in egocentric mental imagery^14^, mental time-travel^15^ and—more generally—reconstructive mechanisms of memory retrieval^16^ and meta-cognitive judgment of one’s memory^17^ (see also the notion of "autonoetic consciousness", proposed by Tulving^18^). Retrieval from the first-person point of view is consistently seen as more detailed, more vivid and associated with greater confidence in the elements recalled^19^, and it has been linked with medial parietal structures such as the precuneus^20^.

Nonetheless, most of previous studies on episodic memory entailed using relatively simple stimuli (e.g. lists of words or static images) and memory for contextual elements was typically reduced to judging simple low-level features, such as the color/position of words presented on the screen^21^, or correspondences between objects and real-world static images^1^. In contrast, the use of more complex and naturalistic stimuli (such as virtual environments, VEs) allows generating encoding experiences that entail active behavior within a coherent and continuous spatio-temporal context, which mimics everyday life experiences^22,23^. Accordingly, VEs allow accounting for two defining characteristics of episodic memory: namely, the storage of events together with their rich and multidimensional spatio-temporal context^24,25^ and the subjective active experience linking the individual and the episode itself^26,27^.

Encoding in realistic VEs has been associated with an increase of memory performance^28^, with improvements related to the sensory details of the environment^29^, the motor engagement of the participants (e.g., active navigation vs. passive transportation)^30,31^ and—more generally—the level of immersivity of the virtual experience (e.g., viewing via head-mounted displays vs. monitors)^32^. In a pioneering imaging study with encoding in VE, Burgess and colleagues^22^ reported retrieval-related activation of a network including medial parietal regions, the hippocampus and parahippocampus (HC/PHC), and several cluster in the prefrontal cortex. More specifically, the authors compared activity when the participants retrieved different elements of the episode (object, place, and person associated with each episode) revealing that the retrieval of the spatial context (i.e. place vs. person) engaged the medial parietal cortex (the precuneus), retrosplenial cortex, parahippocampal gyrus, as well as the lateral prefrontal cortex (see also^33^, for a relevant model of coordinate transformation during memory encoding and retrieval). Beside the possible role in spatial transformations, the availability of multi-dimensional elements defining the encoding context (e.g. what-where-when-who) may contribute to explaining the engagement of the parietal cortex during episodic memory retrieval^21,34^. Classically, the integration of episodic memory elements has been associated with activation and connectivity between the MTL and the medial prefrontal cortex^35,36^. However, the parietal cortex also plays a key role. In particular, Ranganath and Ritchey^37^ proposed a distinction between "two medial memory systems". The Anterior Temporal system (AT, comprising the perirhinal cortex, anterior temporal cortex, lateral prefrontal cortex and amygdale) would be involved primarily in semantics, emotional processing, social cognition and familiarity-based object recognition. By contrast, the Posterior Medial system (PM) comprises the precuneus, the lateral parietal cortex, the parahippocampus, the retrosplenial cortex, as well as the ventro-medial prefrontal cortex. The PM system shares strong connections with the Default Mode Network^38–40^ and, during retrieval, it is thought to instantiate "situational models" integrating rich spatio-temporal contexts and prior knowledge about everyday life situations and environments. This system would play a central role in episodic retrieval and in reconstructive mechanisms thought to underlie the subjective experience of episodic memory retrieval in everyday life^27,41^.

Previous imaging studies that specifically targeted the role of PM/AT during the retrieval of episodic details associated activation of the lateral parietal cortex with the precision of retrieval judgments, while the precuneus correlated with the subjective appraisal of vividness^13,42^. In a more recent study, Cooper and Ritchey^43^ investigated the functional connectivity of the PM/AT systems. The results showed a decrease of network modularity during retrieval as compared with encoding, indicating strengthened inter-network integration during retrieval. In addition, the coupling between the hippocampus and the two networks was found to increase with the increasing precision of the remembered details. The latter indicates that the quality of episodic retrieval doesn't merely rely on the level of activation of specific brain regions, but rather it is best characterized in terms of the dynamic connectivity of the PM/AT systems (see also^44–46^, and^47^ for review).

**Figure 1:**
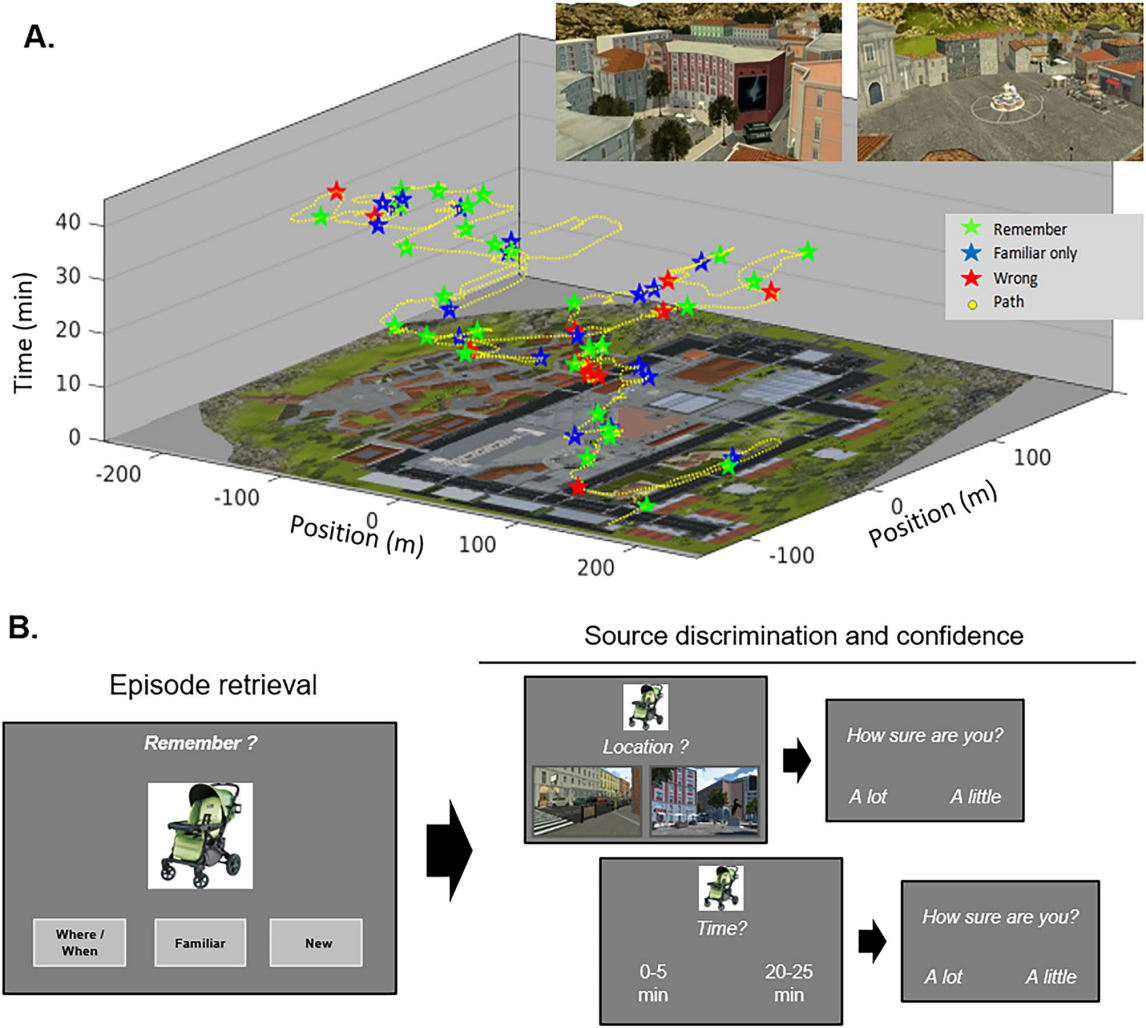
Illustration of the memory encoding and retrieval procedures. (**A**) *Encoding in the virtual town*. Memory encoding took place during 45 min active exploration of a virtual town. The main panel shows a plan of the town (xy-plane) and the path of one participant (yellow dots), with time on the z-axis. During the exploration, the participants were presented with 60 to-be-encoded objects (cf.-colored stars, showing the position and time of these episodes). Each episode is displayed in a different color, as a function of the subsequent responses during memory retrieval. The insets on the top-right show two areal views of the town. (**B**) Retrieval task during fMRI. The memory retrieval was performed during fMRI scanning and included multiple phases. First, in the “episode retrieval” phase, the participants were presented with the image of one object and had to report if: they could remember some spatial or temporal details about the episode (later labeled as *Rem* responses), the object was just familiar (*Fam*), or the object was new (*New*). The analyses of the imaging data targeted activity in this phase of the trial. If the object was old/seen and the participant correctly responded *Rem* or *Fam*, the trial went on with a series of additional questions about the where/when sources. This comprised a place-discrimination test, with the participants having to select out of two the place-images which one corresponded to the place where they had seen the object, and a time-discrimination test involving the choice of the interval when they had seen the object. Confidence judgments (“a little/a lot”) followed each of the source discrimination tests. Place and Time tests were presented in a randomized order across trials. All questions were displayed in French. The images were extracted from the Virtual Environment built with the game engine Unity 3D (http://www.unity3D.com).

The aim of the current study was to extend the investigation of the connectivity between the nodes of the PM memory system by examining the directional (effective) connectivity during the retrieval of naturalistic events. In order to maximize the engagement of "situational models" in the PM system, we used an encoding procedure entailing the exploration of a virtual town, thus comprising events embedded in a coherent spatio-temporal continuum. During a 45 min session, the participants actively explored the virtual town, while carrying out several tasks. At irregular intervals, they were presented with pictures of objects (60 in total) that where later tested in the retrieval session, see Fig. 1A. The retrieval session took place the day after the encoding phase, during fMRI scanning. Each trial started with the presentation of an object (60 trials including "old" objects that were seen during the encoding session and 60 trials depicting "new" objects) and the participants had to report whether: (1) they had seen the object and could remember the place/time of the corresponding event during encoding ("Remember": *Rem*-response); or, (2) the object was "Familiar", but the participants could not remember where/when they saw it (*Fam*-response); or (3) the participants could not remember having seen the object during the encoding (*New*-response).

We operationalized the "subjective sense of episodic recollection" considering the trials when the participants correctly recognized an object that they had seen in the VE and subjectively reported that they could remember the place/time of the event (i.e., *Rem*-responses). Moreover, after each correct object recognition, we included a series of 2-forced-choice discriminations and confidence judgments about the place and the time of encoding (Fig. 1B, see also^48^). The analyses of these source-discrimination tasks enabled us to show that *Rem*-responses did not merely reflect some general effect of memory strength, but they entailed also the availability of context-related information during retrieval. Analyses of effective connectivity using Dynamic Causal Modeling^49^ (DCM) tested specific hypotheses about the modulatory influence of episodic retrieval (i.e. correctly recognized old-items, with *Rem*-responses) on the network's dynamics. To construct the DCM network, we considered areas of the PM system that activated significantly in the intraregional activation analysis that compared correctly recognized old/seen trials with *Rem* vs. *Fam* responses: namely, the left hippocampus/parahippocampus (HC/PHC) in the MTL, the Precuneus (PreC) in the parietal cortex and the ventro-medial prefrontal cortex (vmPFC). In addition, the Fusiform gyrus (FusG) was included as the input node of the network. We tested our main hypothesis about the role of the connectivity between the precuneus and the medial temporal cortex in mediating the subjective quality of retrieval by partitioning the DCM model space in two "Families"^50^. Family 1 included models where *Rem*-responses could modulate the connectivity between PreC and HC/PHC, while in the models of Family 2 this connection could not be modulated by the subjective quality of retrieval. Our main prediction was that models in Family 1 would predict the observed data better than models in Family 2.

## Results

### Retrieval performance

The recognition rate of old/seen objects in the episode-retrieval phase of the trial was 80.9% (standard deviation: std = 10.6) and the response accuracy for the new objects was 70.1% (std = 12.4). For correctly recognized old/seen items, the proportion of *Rem* responses was 73.6% (std = 17.0), with the remaining 26.4% corresponding to *Fam* responses (see also supplementary Table S1, reporting the corresponding number of trails, which were then included in the fMRI analyses). Additional analyses confirmed that overall, the participants could perform object recognition task (d-prime = 1.51, std = 0.49; irrespective of the *Rem*/*Fam* subjective evaluation), but also that the performance associated with *Fam*-responses was not significantly different from chance level (53.3%, std = 16.4, *p* > 0.3, see Supplementary Material). The latter is likely due to the fact that the unseen foil-objects were different exemplars of the same categories of the seen target-objects, hence likely to generate a sense of familiarity and a high rate of false alarms. By contrast, the false alarms rate for trials with *Rem*-responses was low (15.6%, std = 8.7).

**Figure 2:**
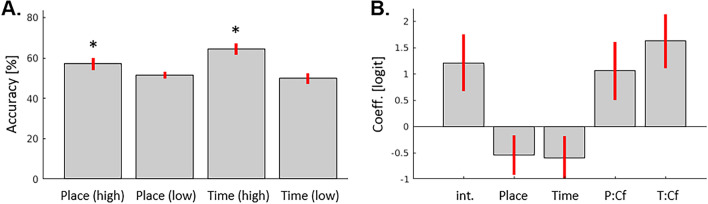
Behavioral results. (**A**) Accuracy of the source-discriminations. Mean accuracy of the Place/Time source discrimination (+ /− sem), as a function of the subsequent confidence rating (“high/low”). Place and Time discrimination followed by high-confidence responses were significantly above chance level (marked with asterisks in the plot), while source discriminations with low-confidence were not significantly different from the 50% chance level. (**B**) Logistic regression. Regression coefficients (logit, ± 95% confidence intervals) of the binomial logistic regression seeking to predict changes of the proportion of *Rem-*responses during episode retrieval using the accuracy and confidence of the subsequent source-memory tests. The model included 5 predictors: the intercept (*int*), the accuracy of the place- and time-discrimination, irrespective of confidence (*Place* and *Time*) and the effect of correct and confident source judgments (*P:CF* and *T:CF*), see also Results section. The results showed a significant contribution of correct source memory on the subjective quality of episode-retrieval, increasing the likelihood of *Rem*-responses specifically when the sources were discriminated with high-confidence (see "P:Cf" and "T:Cf" on the left side of the plot).

The subsequent source-discrimination phase of the trial allowed us to explicitly test memory for the where/when context (see Fig. 1B). Overall, we found that source discrimination was above chance level only when the participants subsequently reported to be confident about their responses ("high" confidence responses). Figure 2A shows the source discrimination accuracy for "Place" and "Time", as a function of high/low confidence (see also Table. S1, for the number of trials contributing to each condition). Place and Time discrimination followed by high-confidence responses were significantly above chance level (one sample t-tests: *p* = 0.037 and *p* < 0.001, respectively), while source discriminations with low-confidence were not significantly different from the 50% chance level (*p* > 0.3 and *p* > 0.9). Additional analyses examined the source-discrimination performance as a function of the subjective quality of the initial episode retrieval response (*Rem* vs. *Fam*), confirming that performance was above chance level for confident source-discriminations that followed episode retrievals with *Rem*-responses (see Supplementary Table S2).

The main aim of the source discrimination phase of the trial was to evaluate whether source-memory contributed to the subjective quality of memory in the episode-retrieval phase of the trial (see also^48^). This was tested using a mixed-effects logistic regression that sought to predict the probability of *Rem*-responses (vs. *Fam*) based on the accuracy and the confidence of the source discrimination. The model included 5 predictors: the intercept, the accuracy of the place- and time-discrimination irrespective of confidence (coding 1/0, for correct/wrong), and the effect of correct and confident source judgments (coding 1 correct source-discrimination followed by high confidence rating and 0 for all the other response combinations). The logistic analysis revealed a significant contribution of correct source memory on episode-retrieval, specifically when the sources were discriminated with high-confidence. Both confident-place and confident-time discriminations lead to an increase of the probability of *Rem-*responses (*p* < 0.001, for the two predictors coding for correct and confident responses; see Fig. 2B, "P: Cf" and "T: Cf" on the left side of the plot). The logistic analysis also indicated that the overall discrimination accuracy, irrespective of confidence, actually lead to a decrease of *Rem* responses (*p* < 0.005 and *p* < 0.004 for Place and Time, respectively; cf. Figure 2B, parameter estimates "Place" and "Time"). The latter may be due to correct "guess-responses" during the source discrimination, followed by low confidence judgments, on trials with *Fam*-responses. Beside this, the results of the logistic analysis confirmed the contribution of confident source-memory to *Rem*-responses during episode retrieval (cf. our previous results using the same encoding, retrieval and analysis procedures in a purely behavioral study^48^).

### Intra-regional activation analyses

The intra-regional analysis aimed at identifying regions that activated during the episode-retrieval phase, specifically when the participants recognized seen/old objects and subjectively felt that they could remember some spatio-temporal detail about the encoding context. For this we directly compared trials with *Rem* vs. *Fam* responses, considering correctly recognized old-trials only. This comparison reveled significant activation in five regions, including the left medial temporal cortex, the precuneus (PreC), the medial prefrontal cortex (mPFC, including a ventral vmPFC cluster and an anterior amPFC cluster), the left middle temporal gyrus (MTG) and a cluster in the left ventral occipital cortex, see Table 1. The cluster in the medial temporal cortex included voxels belonging both the hippocampus (HC) and the parahippocampus (PHC), according to the Harvard–Oxford atlas (https://neurovault.org/collections/262/). More specifically, the activation cluster was located in a region where the Harvard–Oxford probability maps of the HC and PHC overlap. Considering the 18 voxels of the activated cluster, thirteen voxels had higher probability to be in the HC than in the PHC, while 5 voxels had higher PHC probability. Accordingly, we refer to this activation as the hippocampus/parahippocampus cluster (HC/PHC).

**Table 1:** Anatomical locations, *xyz-*peak coordinates in MNI space (Montreal Neurological Institute), and statistical values for the univariate analyses.

	p-FWE-corr	size	x	y	z	Z-value
Remember vs. Familiar (correct old/seen trials only)						
Left HC/PHC	0.001	18	− 24	− 16	− 25	5.45
Precuneus	< 0.001	474	− 6	− 64	26	6.13
medial prefrontal cortex (ventral)	< 0.001	102	6	32	− 19	6.39
Medial prefrontal cortex (anterior)	0.011	12	− 6	56	17	4.91
Left middle temporal gyrus	0.001	122	− 60	− 22	− 13	5.52
Left ventral occipital cortex	< 0.001	352	− 6	− 79	− 7	6.73
Left angular gyrus	*0.019*	*3*	− *57*	− *55*	*20*	*4.77*
Retrosplenial cortex	*0.024*	*3*	*12*	− *52*	*20*	*4.72*
Old vs. New (correct trials only)						
Precuneus	0.002	58	− 18	− 58	20	5.32
Left ventral occipital cortex	< 0.001	150	− 6	− 82	− 7	5.86
Left anterior IPS	0.004	22	− 33	− 52	41	5.00
Left cerebellum (*)	< 0.001	29	− 15	− 55	− 16	5.60
Right precentral gyrus	0.006	82	42	− 13	62	5.05
Interaction old/new by accuracy						
Left anterior IPS	0.006	11	− 48	− 43	50	5.02
Left ventral occipital cortex	0.001	85	− 6	− 79	− 7	5.52
Left cerebellum (*)	< 0.001	67	− 18	− 55	− 13	5.75

*p-FWE-corr* values are corrected for multiple comparisons at the voxel-level considering the whole brain as the volume of interest (minimum cluster size = 10 voxels). For the "remember vs. familiar" contrast the table includes two additional clusters (i.e. left angular gyrus and retrosplenial cortex, in italics) that did not pass the minimum cluster size threshold, but are reported here because they are part of the PM system. *Size* in voxels. HC/PHC: hippocampus/parahippocampus; IPS: intraparietal sulcus. (*) The peak activation was located in the cerebellum, but the clusters extended dorsally into the adjacent ventral occipital cortex.

**Figure 3:**
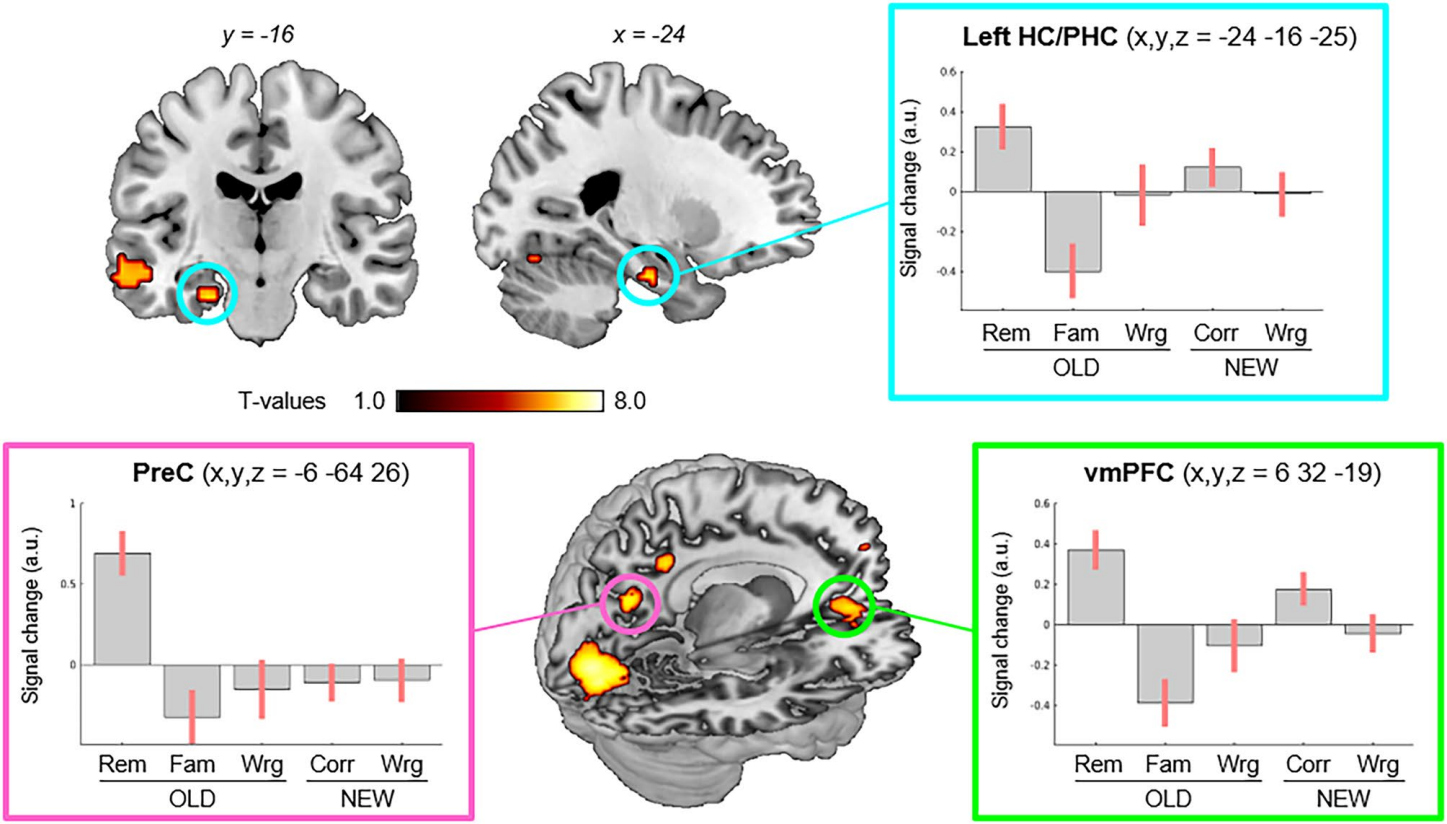
Remembered objects. Intra-regional activations associated with *Rem* responses, as compared with *Fam,* during the episode retrieval (cf. bar 1 vs bar 2, in each plot). This comparison showed activation of the left hippocampus/parahippocampus (HC/PHC, top panel, in cyan), the precuneus (PreC, in magenta) and the ventro-medial prefrontal cortex (vmPFC, in green). The same comparison also showed significant activation of the left lateral temporal cortex (visible in the top-left coronal section) and the occipital cortex (visible in the 3D rendering). Activations are displayed at p-FWE-corr = 0.05, corrected for multiple comparisons at the whole brain level (T-value = 4.9, min. cluster size = 10 voxels). Rendering was done using MRIcron^51^. The signal plots show the parameter estimates at the peak-voxel and for all the episode-retrieval conditions. These include correctly recognized new-items (“NEW Corr”) and incorrect responses (“Wrg”, plotted separately for old/seen and new/unseen trials). The parameter estimates of the general linear model are mean-adjusted (sum = 0) and negative values should not be interpreted as de-activations.

Figure 3 shows the anatomical location of the areas activated for the *Rem* vs. *Fam* comparison and the signal plot of the maxima in the left HC/PHC, the PreC and vmPFC that belong to the PM memory system^37^. The plots highlight greater activity during correct old-trials with *Rem*-responses (bar 1) vs *Fam*-responses (bar 2) in all three areas. The signal plots also show the level of activation in the other 3 episode-retrieval conditions (*OldErr*, *New* and *NewErr*) indicating that in the PreC the activation was highly specific for the *Rem* condition (bar 1). A similar pattern of activation was found also in the left angular gyrus (AG) and the retrosplenial cortex (RSC), even though these two clusters were too small (3 voxels) to pass our statistical threshold (minimum cluster size = 10 voxels; see Table 1). Because both these regions are part of the PM system, their location and signal plot are reported in Supplementary Figure S1 that also highlights the separation between the *Rem*-specific activation in the left AG versus the overall effect of correct object-recognition in left intraparietal sulcus (see also below).

Together with the main "*Rem* vs *Fam*" comparison, we performed additional contrasts to identify other regions involved in the episode retrieval. We tested for areas that activated during the correct retrieval of old/seen objects compared with new objects, irrespective of subjective memory. The corresponding contrast ("*Rem* + *Fam* vs *New*", correct trials only) reveled again activation of the PreC and the left ventral occipital cortex, as well as the left anterior intraparietal-sulcus (aIPS), plus two clusters located in the left cerebellum and the right precentral gyrus, see Table 1. We asked to what extent these activations reflected specifically the successful retrieval of seen/old objects by testing for the interaction between old/new-objects and response accuracy. This showed significant effects in the left aIPS, the left ventral occipital cortex and the cerebellar cluster (Table 1, and Supplementary Figure S1).

### Inter-regional effective connectivity (DCM)

The main aim of the current study was to investigate the effective connectivity of the PM system during the retrieval of naturalistic episodes. In particular, we targeted the role of the connectivity between the PreC and the medial temporal cortex in mediating the effect of subjective sense recollection (*Rem*-responses). For this we considered the regions of the PM system that activated for the "*Rem* vs. *Fam*" contrast (PreC, HC/PHC and vmPFC, cf. above and Fig. 3) and constructed two Families of DCM models^50^: Family 1 included models where *Rem*-responses were allowed to modulate the connectivity between the PreC and HC/PHC; and Family 2 that included models without this modulatory effect. Within each Family, the different models allowed us to test more globally the effect of *Rem*-responses on the connectivity between the input region (FusG) and PreC/vmPFC and of the HC/PHC-vmPFC connection (see Supplementary Figure S2, showing the whole DCM model space).

**Figure 4:**
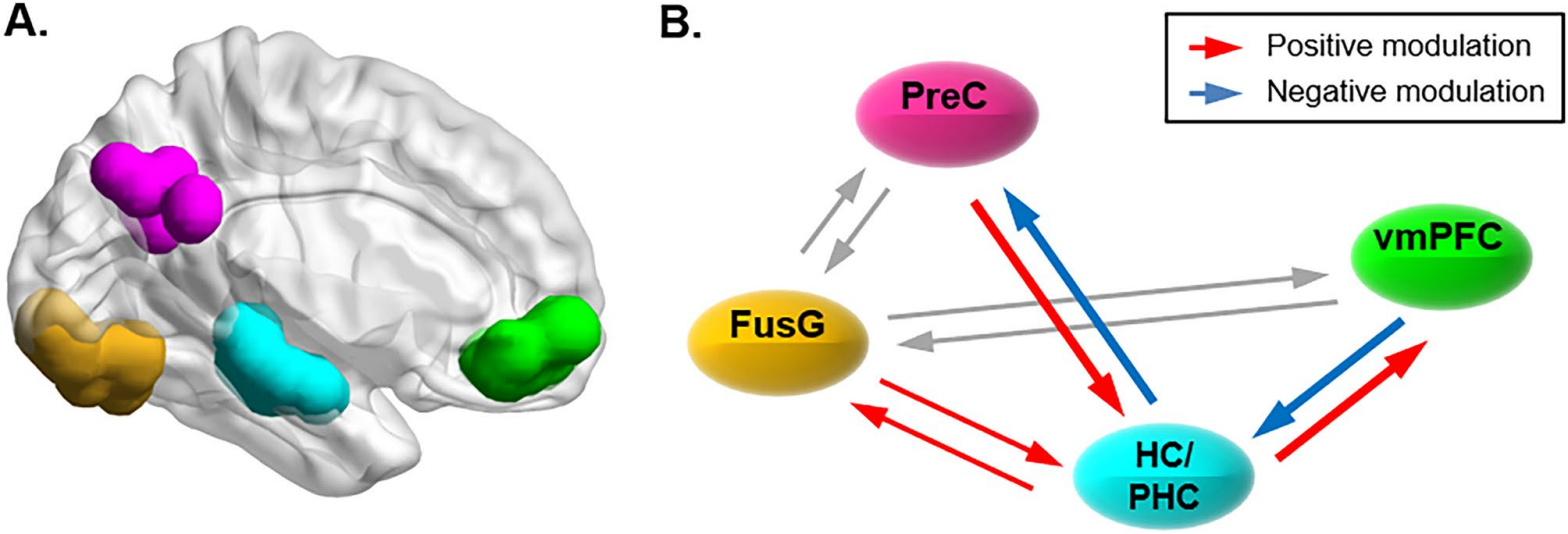
Individual ROIs and dynamic causal modeling. (**A**) Regions of Interest. The individual ROIs projected on a 3D rendering of the left hemisphere, with different colors for the Precuneus (PreC, in magenta), the left fusiform gyrus (FusG, orange), the left hippocampus/parahippocampus (HC/PHC, cyan) and the ventro-medial prefrontal cortex (vmPCF, green). The ROIs comprised 8 mm-radius spheres defined on the basis of a combination of functional and anatomical criteria (see Methods section). These ROIs were used for the analyses of effective connectivity (DCM: see panel B). The 3D rendering was produced using BrainNet^53^. (**B**) Connectivity modulation by Rem-responses. Results of the analyses of effective connectivity using Dynamic Causal Modeling. The panel shows the model that best explained our data according to Bayesian Model Selection (see also Supplementary Figure S2, “Mod_2”). The winning model included modulatory influences of the *Rem* condition on the connectivity of the medial temporal cortex (HC/PHC) both with the precuneus and the ventro-medial prefrontal cortex. By contrast, the connections between the fusiform gyrus (FusG, the input node of the model) and the PreC/vmPFC were unaffected (gray arrows). Bayesian Parameter Averaging of the winning model showed that the *Rem* condition had opposite patterns of positive/negative modulation on the HC/PHC-PreC and HC/PHC-vmPFC connections (see red/blue arrows: posterior probability > 99%), as well as highlighting the effect of *Rem* of the FusG-HC/PHC connectivity. The images were made with Statistical Parametric Mapping software SPM12 (Wellcome Department of Imaging Neuroscience, University College London, UK; http://www.fil.ion.ucl.ac.uk/spm).

Bayesian model selection^52^ (BMS) showed that Family 1, which included the modulation of the PreC-HC/PHC connection, was most likely to explain our data (> 99% posterior probability), strongly supporting the hypothesis of a contribution of the PreC-HC/PHC connectivity during episodic memory retrieval with subjective sense of recollection. At the model level, BMS showed that the model with the highest posterior probability (> 99%) included the modulation of the PreC-HC/PHC, as well as the modulation of the vmPFC-HC/PHC connection, indicating that both regions of the PM system contributed to *Rem* retrievals (see Fig. 4B, corresponding to mod_2 in Supplementary Figure S2).

We examined the connectivity of the winning model using Bayesian Parameter Averaging^54^ (BPA) and assessed the posterior probability of the modulatory influences associated with the *Rem* condition. In agreement with the BMS results, we found that *Rem* modulated the connectivity between the PreC-HC/PHC and vmPFC-HC/PHC with posterior probabilities > 99%. This included the connections from HC/PHC to PreC and vmPFC, and the connection from PreC and vmPFC to HC/PHC. However, the signs of these bidirectional effects were different for the two regions: *Rem* made the influence of PreC-to-HC/PHC faster (positive parameters) and the influence of HC/PHC-to-PreC slower (negative parameters); while the opposite pattern was found between HC/PHC and vmPFC, see Fig. 4B. The BPA also revealed positive effects of *Rem* on the bidirectional connection between FusG and HC/PHC (posterior probabilities > 99%, Fig. 4B).

For the winning model we also examined the underlying intrinsic connectivity (A-parameters, cf. Methods section) and the effect of the driving inputs (C-parameters). The analysis of the intrinsic connectivity confirmed significant couplings between the nodes of the network, see Figure. S3 (right panel). The coupling between the FusG and both HC/PHC and PreC were positive in both directions. By contrast the coupling between the HC/PHC and PreC exhibited a pattern opposite to that observed for the modulation by *Rem* : that is, HC/PHC had an intrinsic driving effect on PreC, while the influence of PreC on HC/PHC was negative (compare in Fig. 4B and Figure. S3, right panel). Accordingly, memory retrieval associated with a subjective sense of recollection entailed modulatory influences opposing the intrinsic coupling between the hippocampus and the precuneus, see also “Discussion” section.

As noted above, by dropping the minimum cluster size criterion in the intra-regional *Rem* vs *Fam* activation analysis, we also found activation of the left AG and the RSC that are part of the PM system (see also Table 1). We sought to explore this further by carrying out an analysis of effective connectivity that now included also these two regions. The inclusion of these additional areas in the model leads to an huge increase of the number of possible routes for signals to flow between the PreC and the medial temporal cortex (i.e. direct connection, connection via FusG, via AG, via RSC or via various combinations of these). This makes it difficult to use family/model selection to test specific hypotheses about our main experimental question. Accordingly for this exploratory DCM analysis we made use of Parametric Empirical Bayes (PEB)^55^ and Bayesian Model Reduction (BMR)^56^. We constructed a fully connected DCM and allowed *Rem*-responses to modulate all the connections. We then used the function "search over nested PEB models" that iteratively removes any parameters that do not contribute to the model evidence and averaged the models from the last iteration using Bayesian model averaging^55^. The resulting model displayed several differences compared to the main DCM analysis (e.g. now the negative effects on HC/PHC arose primarily from AG and RSC, rather than from vmPFC), but—most important—also this fully explorative approach highlighted a positive effect of *Rem*-responses on the connection from the PreC to the HC/PHC (see Supplementary Figure S4).

## Discussion

We investigated the retrieval of information encoded in a rich, "life-like" virtual environment asking how the subjective quality of retrieval impacted on the directional connectivity between the medial parietal cortex and the medial temporal cortex. Intra-regional analyses revealed that item-retrieval associated with the subjective sense of recollecting the contextual details of the encoded episode activated the precuneus in the medial parietal cortex, the hippocampus/parahippocampus in the medial temporal cortex and the ventro-medial frontal cortex ("*Rem* vs *Fam*"; see Fig. 3). By contrast, the left intraparietal sulcus activated for correct recognition of old/seen items, irrespective of subjective evaluation. The relevance of contextual information in contributing to the subjective sense of episodic retrieval was confirmed at the behavioural level, with logistic regressions showing that the likelihood of *Rem*-responses increased when participants were able to discriminate with high-confidence the place/time of the event in subsequent forced-choice tests (Fig. 2, see also^48^). Analyses of effective connectivity allowed us to confirm the functional coupling between precuneus and the medial temporal cortex, here showing for the first time that the subjective sense of recollection modulates this coupling, with an increased influence of PreC on HC/PHC during the retrieval of episodes with the associated spatio-temporal details (Fig. 4). Our results contribute to delineate the role of the medial parietal cortex during the retrieval of complex memory traces, demonstrating that the precuneus exerts an influence on the medial temporal cortex specifically during the retrieval of memories entailing episodic qualities.

The hippocampus/parahippocampus, the precuneus and the ventro-medial prefrontal cortex are part of the posterior medial memory system^37^ (PM) and their activations are consistent with previous imaging studies of episodic retrieval following encoding in life-like virtual environments^22,57^. The left angular gyrus and the retrosplenial cortex are also part of this system and here were found to activate just below threshold (see Supplementary Figure S1). The PM system has been associated with the representation of "situational models" that would provide a scaffolding knowledge about spatio-temporal relationships, which would support reconstructive processes^16,37^.

Here we found a dissociation between the activation of precuneus (and the angular gyrus in the ventro-lateral parietal cortex) that activated selectively for the "*Rem* vs. *Fam*" comparison and the left intraparietal sulcus that instead activated during correct recognition of old/seen objects, irrespective of the subjective quality of the retrieval (see Table 1 and Supplementary Figure S1). This dissociation is in agreement with previous studies that associated the activation of the ventro-lateral parietal cortex, and in particular of the left angular gyrus, with the representation memories retrieved with high-levels of confidence and/or rich details^13,58,59^, versus overall effects of accurate retrieval more dorsally in the parietal cortex^60^. Possible dissociations between dorsal versus ventral parietal regions during memory retrieval have been largely debated in the literature. In particular the AtoM-model^61^ suggests that dorsal regions may engage then strategic top-down control mediates retrieval, while ventral regions would engage primarily when memories are retrieved in a bottom-up, automatic manner. In this framework, we could speculate that the presentation of the object-image in episode retrieval phase triggered bottom-up mechanisms of retrieval (and activation of the ventral, plus medial parietal cortex), while the engagement of the intraparietal sulcus may reflect the engagement of endogenous memory search, irrespective of whether this led to the successful retrieval of contextual details. The use of alternative retrieval strategies (e.g. by using verbal cues, rather than images) could help disentangling the relative contribution of endogenous vs. exogenous factors, albeit such changes in the mode of retrieval would most likely entail the engagement of other brain regions (e.g. additional frontal areas, when accessing memory in a fully endogenous manner, e.g. via free recall^62^).

Here we found activation of the precuneus in the medial parietal cortex when the participants subjectively reported that they could remember contextual elements of the episode (*Rem*-responses). This extends previous findings that associated activation of the precuneus with the subjective quality of episodic retrieval, such as the vividness of rich memories^13,42^. In Richter's study^13^, following the encoding of static pictures, the retrieval tasks quantified the level of precision and vividness of the memories and revealed a dissociation between retrieval success in the hippocampus, precision in the lateral parietal cortex (AG) and vividness in the precuneus. Here the pattern of activation in the PreC and the left AG were alike (cf. Fig. 3 and Supplementary Figure S1), albeit the effect of *Rem* vs *Fam* was fully significant in PreC only. A main difference between Richter's study^13^ and the current protocol is that while Richter's study sought to obtain objective measures about the retrieved information, here we focussed on the subjective evaluation of the retrieval. The latter may entail the appraisal of a combination of factors, including the precision and vividness of the retrieved information, as well as the overall memory strength.

Related, it should be noticed that use of *Rem*/*Fam* judgements to evaluate the subjective sense of episodic recollection has been questioned by several authors^63,64^. Remember/Know judgments aim to disentangle retrieval based on recollection as opposed to familiarity in the framework of dual-processes models of episodic memory^65^, with recollection also entailing the sense of reliving the encoding experience^18^. Nonetheless, there is evidence that "remember" vs. "know" responses may rely on different strengths of memory within a signal-detection framework^63,66^ (but see also^67^, suggesting a possible dissociation between the evaluation of recall, subjective experience and confidence). Here we used the dichotomic *Rem*/*Fam* judgments to operationalize the notion of subjective evaluation of episode retrieval, but we acknowledge that the subjects' responses may also reflect different memory strengths (and/or subjective confidence). However, the inclusion of the additional source-discrimination tasks enabled us to demonstrate that *Rem*-responses entailed the availability of some information about the spatio-temporal context and did not solely rely on memory confidence/strength (but see also^68^). The results of the logistic regression analyses (Fig. 2B) showed that there was an increase likelihood of *Rem*-responses when the participants were correct and confident about the subsequent source discriminations. The behavioural data also indicated that subjective confidence about source-information played a major role in the current task, as evidenced by the poor performance associated both with *Fam*-responses in the episode retrieval phase and with low-confidence judgements during source discriminations (see also Supplementary Tables S1–S2). The difficulty of retrieving specific source-information was possibly due to the fact that the participants encoded the objects in an incidental manner, that the first-person views of the town used for the place-discrimination were visually quite similar to each other (because of the use of a limited number of rendering materials) and that the time-judgments involved selecting from a relatively large number of nine different temporal intervals. The overall difficulty to select and retrieve highly related contextual information may also explain the activation of areas in the visual and the lateral temporal cortex that do not belong to the PM system. The activation of occipital visual regions may reflect the active maintenance of visual information during episode retrieval^69^ (here, the visual details of the spatial context) and the lateral temporal cortex may contribute to relational/associative processes, combining episode-specific information with more general "semantisized" knowledge about the visual appearance of the virtual town^70,71^ (see also^64,72^).

Beside the activation of specific brain regions, the primary objective of the current study was to characterise the dynamic causal interactions between the precuneus and the medial temporal cortex, as a function of the subjective quality of the retrieved episode. We constructed DCMs considering the areas of the PM system that showed a significant activation in the univariate activation analysis (i.e. the precuneus, the left HC/PHC and the vmPFC) and assessed changes of connectivity associated with the *Rem*-responses. Hypothesising that the precuneus plays a key role during the retrieval of episodes encoded within rich and structured contexts (cf. representation of "situational models"^37^; see also^25,73,74^), we compared a set of models where the *Rem*-condition modulated the connection between PreC and HC/PHC with a set that did not include this modulatory effect (DCMs Family 1 vs. Family 2, see Supplementary Figure S2). The results showed that the first set of models was most likely to account for the observed data, demonstrating that the PreC-HC/PHC connectivity mediates the subjective quality of retrieval. Further, results at the single model-level revealed that, together with the PreC-HC/PHC connectivity, the *Rem*-condition also modulated the connectivity between the medial temporal cortex and the ventro-medial prefrontal cortex (vmPFC), as well as the HC/PHC and the fusiform gyrus.

Our current findings that the subjective quality of memory retrieval modulates the connectivity between the nodes of the medial memory system complement previous work that sought to understand memory retrieval in a dynamic network perspective (see^75,76^, for recent reviews). For example, using simple stimuli (lists of words), Geib and colleagues^46^ reported changes of inter-regional connectivity as a function of retrieval success. The results highlighted a stronger coupling of the left hippocampus with the precuneus, the lateral parietal cortex and the superior frontal gyrus, specifically when the participants correctly retrieved old words and did so with a high-level of confidence. Mendelsohn and colleagues^44^ reported related effects of retrieval accuracy on functional connectivity, but now during the retrieval of more complex and naturalistic material. Following the viewing of a 45-min documentary, the participants evaluated a set of statements about the movie (true/false, plus judgment confidence) under fMRI. The pattern of activation associated with memory retrieval highlighted the involvement of areas also observed in the current study, including the hippocampus, precuneus and vmPFC and the lateral temporal cortex, plus the left inferior frontal cortex, lateral parietal cortex and the dorso-medial PFC. Analyses of functional connectivity associated correct behavioural performance with an increased correlation between activity in the left hippocampus and the temporal/parietal cortices. In the framework of the PM/AT systems, using static images Cooper and colleagues^43^ showed that the precision of the retrieval modulated both the intra- and inter-network connectivity of the AT and the PM, including the precuneus, as well as the connectivity between these two systems and the hippocampus.

Thus, consistent with our findings here, previous studies highlighted the relevance of the connectivity between the medial temporal cortex and the precuneus for memory retrieval and any modulation thereof by performance, type of retrieved information and the subjective quality of retrieval. Nonetheless, these previous works employed methodologies that rely primarily on examining patterns of correlation between the fMRI time-series, which prevents any inference about the directonality/causality underlining the observed changes (albeit note that Cooper's study^43^ reported psycho-physiological interactions seeding these analyses both in the hippocampus and the PM/AT systems, and thus obtaining separate connectivity parameters for the two directions). Here, we made use of DCM and Bayesian model selection to test hypotheses about the pattern of effective connectivity most likely to explain the observed data. Further Bayesian parameter averaging allowed us to assess the directional/causal structure of the winning model. This revealed that irrespective of condition (intrinsic connectivity, see Figure. S3), the HC/PHC exerted a positive effect on the precuneus, with a negative influence in the opposite direction. Notably, the condition-specific modulatory effect of subjective recollection (*Rem*-condition) was found to counteract this pattern reducing the influence of HC/PHC on PreC and augmenting the causal influence of the precuneus on the medial temporal cortex (Fig. 4B).

The current finding that the subjective quality of the retrieval modulates the effective connectivity between HC/PHC and PreC parallels the work of Ren and colleagues^77^, albeit the directionally of the relevant effects appear to differ in the two studies. Ren's study^77^ investigated involuntary memory retrieval during news-clips watching. The main comparison considered activity/connectivity while the participants watched the second part of a clip (thus, enabling them to process that new information with respect to any information that they had encoded while watching the first part, i.e. "involuntary retrieval") versus watching an unrelated/new clip (i.e.: a "no retrieval", control condition). The authors constructed DCMs comprising the hippocampus, the precuneus and the angular gyrus (plus, the visual cortex, as the DCM input node, as here) and showed that memory retrieval resulted in an increased connectivity between the precuneus and the AG, the hippocampus and the AG and bidirectionally between the hippocampus and the PreC. Most relevant, they also reported that the directional connection between the medial temporal cortex and PreC correlated with individual confidence in their memory (but not with accuracy). The result appears thus opposite to our current findings highlighting a driving effect of the PreC on the HC/PHC associated with the *Rem*-condition. Nonetheless, it should be noted that in Ren's study^77^, the relationship between brain connectivity and subjective memory was assessed simply by correlating the intrinsic network parameters with behavioural data. By contrast, here we specifically modelled the modulatory effect of the *Rem*-condition in the DCMs, thus explicitly accounting for the impact of the behavioural outcome on the network dynamics. Moreover, Ren's study^77^ targeted involuntary/automatic retrieval using a task-free procedure building on narratives' coherence between the "encoding" and the "retrieval" phase. By contrast, our procedure entailed the presentation of specific objects during encoding and a strategic/voluntary access to contextual details in the retrieval phase. These differences are likely to impact on the balance between bottom-up vs. endogenous processes engaging during retrieval (see also above), and—in turns—on the dynamics of the retrieval network. Moreover, it should be noticed that while in our study the test-objects were presented within a rich and dynamic context, these were not part of the virtual environment and were not relevant for the participants' task/missions. This choice relates to the fact that the current encoding-retrieval procedure was designed to be suitable also for object-encoding in the real world (see^48^). Future studies should directly address how the level of action/interaction between the participant and the to-be-remember objects presented in naturalistic conditions impacts of subsequent memory for the objects and for their spatio-temporal encoding context.

Together with the PreC-HC/PHC connectivity, our main DCM analysis indicated that the subjective feeling of recollection modulates the connectivity between the HC/PHC and the vmPFC. The latter is also part of the PM system and showed a significant intra-regional activation for the "*Rem* vs *Fam*" comparison (see Fig. 3). In terms of effective connectivity, the analysis of the parameters of the winning model revealed a driving effect of *Rem*-responses on the HC/PHC-to-vmPFC connection. Note that, somewhat analogous to the HC/PHC-PreC connection, also here the modulatory effect of subjective memory quality was opposite to the pattern of intrinsic connectivity (see Fig. S3, with a negative effect of HC/PHC-to-vmPFC). Using DCM, St. Jacques and colleagues^78^ reported that a medial temporal network exerted a positive effect on a medial PFC network during memory search/construction. This relationship was then inverted when the participants were asked to elaborate the retrieved material. The inversion of the directionality of the connectivity between the medial temporal cortex and mPFC seems to match our observations here (cf. intrinsic connectivity vs. modulatory effects) and may reflect different stages of the retrieval. This would entail an effect of mPFC to the medial temporal cortex during memory search, versus signalling from the medial temporal cortex to mPFC when the relevant information about the episode is evaluated (but see^79^, reporting a directional influence of vmPFC on the medial temporal cortex also during "elaboration").

Despite some discrepancies between studies, which may reflect differences in terms of paradigms (simple vs. naturalistic encoding condition vs. autobiographic memory, which would entail different levels of competition between the stored information; e.g. see^57^) as well as methodological aspects (incl. the regions considered for the analyses of connectivity; e.g., dorsal vs. ventral mPFC), the investigations of the dynamic interactions between the medial temporal cortex, the parietal cortex and the PFC suggest that these connections may engage multiple times during episodic memory retrieval (see also^80^). We propose that in our paradigm the retrieval of episodes entailed at least two phases. One phase would comprise searching for the relevant episode, with the medial temporal cortex combining item-context information^36,76^ and providing any such (candidate) episode-specific information to "situational models" in precuneus^37^. The latter would enable apprising the episode (i.e. the cued object and the candidate place/time) in the context of the global spatio-temporal representation of the 45-min experience in the virtual environment. In our DCM results these processes would be expressed at the level of the intrinsic connectivity (i.e., independent of the subjective sense of recollection, Figure. S3). A second phase would specifically relate to the retrieval of the episode together with its contextual place/time dimensions (*Rem*-condition). The latter would entail the modulation of the connectivity between PreC-HC/PHC-vmPFC, now with PreC driving the HC/PHC and HC/PHC driving the vmPFC, Fig. 4). Nonetheless, we should point out that these effects entailing both the PreC-HC/PHC and the HC/PHC-vmPFC connectivity were observed only in our main DCM analysis that included a restricted number of regions and that embodied a set of a-priori hypotheses about the impact of *Rem*-responses on the network dynamics (cf. "Families of models" in Figure. S2). Complementary analyses of effective connectivity that considered additional regions (i.e. the left angular gyrus and the retrosplenial cortex) and that explored the effect of *Rem*-responses on all possible combinations of connections, replicated the influence of the PreC on the HC/PHC, but now highlighted the importance of the connectivity between AG/RSC and HC/PHC, rather than HC/PHC-vmPFC (see Supplementary Figure S4).

In conclusion, we showed that the successful retrieval of events encoded during active exploration of a complex virtual environment activated regions of the medial posterior memory system (HC/PHC, PreC and vmPFC), selectivity when the retrieval was accompanied by the subjective sense of remembering the spatio-temporal context of the events ("*Rem* vs. *Fam*" responses, Fig. 3). Behaviourally, we found that the likelihood of *Rem*-responses increased when the participants were subsequently able to correctly and confidently discriminate the place/time of the event. The latter indicates that *Rem*-responses were associated with the availability of some spatio-temporal details about the encoded episode, beyond any overall effect of memory strength (Fig. 2; see also^48^). Most important, the results of the analyses of effective connectivity supported our main prediction that the subjective quality of retrieval entailed a modulation of the connectivity between the precuneus and the medial temporal cortex (Fig. 4). The analyses of the directionality of these effects and of the pattern of intrinsic connectivity let us to propose that here episodic retrieval entailed two main phases: (1) signalling from the medial temporal cortex to the precuneus mediating the search of the to-be-remembered event, supported by "situational models" that represent the rich encoding experience; and (2) transient signalling in the opposite direction tagging the successful retrieval of the event together with its spatio-temporal context. We conclude that the subjective quality of episodic retrieval is best understood considering the network's dynamics within the PM system (see also) and that the driving influence of situational models (represented in the precuneus) on the medial temporal cortex determines the subjective sense of episodic recollection.

## Methods

### Participants

Twenty right-handed healthy adults were recruited for the study. One participant could not complete the study because of technical problems with the system controlling the experience in the virtual environment, leaving 19 participants for data analysis (mean age: 28.2 [std = 3.9], range 23–35, 6/13 males/females). The sample size was chosen based on our previous work showing reliable activation of the precuneus during the retrieval of complex, naturalistic material^73^, and a behavioral study that used the same encoding/retrieval procedures as here^48^. Nonetheless, we acknowledge the relatively small sample size is a limitation of the current study. The participants had normal or corrected-to-normal vision, no neurological, psychiatric or cognitive impairments and gave their written informed consent to participate in the study. The study was approved by the Committee for Person Protection (CPP OUEST II, France, A02558-45), in accordance with the Declaration of Helsinki.

### Experimental procedures

The experimental protocol involved two sessions on two subsequent days. The first day the participants encoded a set of 60 memory items (pictures of objects), while actively exploring a large-scale virtual town. On the following day, during fMRI scanning, they performed a retrieval task assessing the subjective memory status of the encoded objects (Remembered, Familiar, or New), as well as memory and confidence for the what/place and when/time elements of the episodes (see Fig. 1).

#### Encoding session

During the encoding session, the participants sat in front to a PC screen and actively explored a virtual town using the PC-keyboard (see Fig. 1A). The virtual environment was built with the game engine Unity 3D (http://www.unity3D.com). The town comprised an area of 380 × 280 m and included approx. 100 shops, a large number of avatars (ca. 250), a dozen moving cars and many environmental sounds. Over a period of 45 min, the participants walked through the town with the aim of completing a set of "missions". Each mission required the participants to collect a specific object (e.g. a packet of cigarettes, a T-shirt, etc.) that could be found in the corresponding shops (i.e. tobacconist, clothes-shop, etc.). There was a total of 15 possible missions. Each mission was triggered when the participant visited a specific location of the virtual town. Accordingly, the individual exploration-path determined whether or not a given mission was triggered. When the mission was triggered, the participant received the instruction to collect a specific object, either via interactions with an avatar who gave verbal instructions or via a virtual "phone call" comprising a ringing tone, followed by a voice giving the instructions. On average across participants, the number of triggered missions was 9.6 (std = 1.9, max = 13). Out of these, 8.2 missions were completed on average (std = 2.1, max = 11). Before starting the session, the participants also received a list of 5 additional objects that they had to collect ("on-going" missions). The participants could enter in any shop and collect objects at any time, but received 20 points only when they collected a currently relevant (instructed) object. If they collected an irrelevant object, 10 points were subtracted out. As soon as a relevant object was collected, it became irrelevant. At any moment, the participants could check an "inventory-screen" reporting the time elapsed from the start of the experience, the list of the 5 initial objects (on-going mission) and the number of points.

At unpredictable times, the navigation was stopped, there was a ringing tone and the picture of a to-be-encoded object was displayed in the lower part of the screen. The task of the participants was to report whether they liked/disliked the object using the PC-keyboard. The like/dislike judgment was included to make sure that the participants paid attention to the memory-objects. During the 45 min session there were 60 of these events, comprising 60 different objects. The pictures of the objects were taken from the Massive Memory Object Categories database^81^ and included common objects of different categories (e.g. tools, animals, food). The presentation order of the objects was fixed for all the participants, but the location where these events occurred was specific for each participant depending on their exploration path. The timing of the events was also approximately the same for all subjects, but could slightly vary depending on the participants behavior. Before presenting the memory-object, there was a verification that the participants were not receiving any instruction and that they were not collecting any object in the shops. In these cases, the presentation of the memory-object was delayed of a few seconds, which lead to some variability in the timing of the events across participants.

#### Retrieval session

The retrieval session took place the day after the encoding phase, during fMRI scanning. The retrieval task comprised 120 trials: 60 trials including "old" objects that were seen during the encoding session and 60 trials depicting "new" objects. The "new" objects were selected to be from the same categories as the seen/old objects, making the object-recognition judgment relatively difficult. Old and new trials were randomized and presented in two separate fMRI runs, each including 60 trials. The memory task entailed an "episode retrieval" phase and, for correctly recognized old items, a "source discrimination" phase (see Fig. 1B). The analyses of the imaging data targeted the episode retrieval phase, while the behavioral responses for the source discrimination served to evaluate the contribution of place/time memory to the subjective memory status of the object at episode retrieval (see "Data analysis: behavior", below).

During the episode retrieval phase, the participants were presented with the picture of one object and they were asked to report: (a) I have seen this object and I have some memory of the place/time when this happened; (b) The object is familiar, but cannot remember when/where I saw it; (c) I have not seen the object. We label these three response-types "Remembered" (*Rem*), "Familiar" (*Fam*) and "New" (*New*). The participants responded using an MR-compatible button-box. The image of the object remained on the screen for a variable interval between 6 and 8 s. If the object was seen during encoding and the participant responded *Rem* or *Fam*, the trial went on testing for source memory. If the object was not seen during encoding or the participant responded *New* to a old object, the trial was terminated.

The source-memory test included 2-forced-choice discriminations about the episode's place and time, plus corresponding confidence judgments. The place-test consisted in the presentation of two place-images shown side-by-side (see Fig. 1B). Both images were recorded during the encoding session of each participant and thus corresponded to individual first-person views of the environment. One of the two images (the target) was taken when the memory-object event was presented, while the second image was not associated with any memory-object event. The participants had to respond which image corresponded to the place where they had seen the object. The two images were displayed for 6 s, together with the memory-object and the written instruction: "What place?". Next, the question "Are you sure (place)?" appeared on the screen and the participants had to choose between the two responses: "little" vs. "a lot". The question stayed on screen for 4 s. The time-test had an analogous structure. First, two time-intervals were presented to the participants, one including the moment when the memory-object was presented during the encoding (i.e. the target-interval). There were 9 possible intervals, each comprising a 5 min duration (i.e. 0–5 min; 5–10 min, etc.). The foil-interval was never contiguous to the target interval and it included the time when the foil place-image was taken (cf. place-test). The participants had to report which interval included the time when they had seen the memory object. This was followed by the confidence question "Are you sure (time)?". The order of the place/time source-tests was randomized within participants. The inter-trial interval was 3–5 s.

### fMRI image acquisition and preprocessing

T2*-weighted echo-planar images (EPI) with blood oxygen level-dependent (BOLD) contrast were obtained using a 3T MRI System (Trio, Siemens: 40 slices covering the entire brain, Repetition Time = 2.2 s, echo time = 30 ms, voxel size = 3 × 3 in plane; slice thickness = 2.5 mm; gap = 20%). Each participant underwent 2 functional imaging runs. The duration of each run was variable, depending on the number trials that the participant correctly recognized as "old/seen" thus triggering the additional series of source discrimination questions (range of volumes: 952–1145).

The BOLD images were preprocessed and analyzed with Statistical Parametric Mapping software SPM12 (Wellcome Department of Imaging Neuroscience, University College London, UK; http://www.fil.ion.ucl.ac.uk/spm). After discarding the four first volumes of each fMRI-run, images were corrected for head movements. Slice-acquisition delays were corrected using the middle slice as reference. All images were normalized to the SPM12 Tissue Probability Map and re-sampled to 2 mm isotropic voxel size. The data were spatially smoothed using an isotropic Gaussian kernel of 8 mm full-width at half-maximum (FWHM).

### Data analysis

#### Behavior

Following the procedure that we employed in a previous behavioral study using the same paradigm^48^, the main analysis of the behavioral data sought to confirm that source memory contributes to the subjective memory status of the encoded objects: namely that correct and confident place/time discrimination is associated with an increase of the likelihood to recognize old/seen objects as "Remembered", as opposed to just "Familiar". For this, we used a mixed-effect binomial logistic regression. The model considered all trials when an old/seen-object was correctly recognized either as *Rem* or *Fam*. The dependent variable was the *Rem* or *Fam* memory status of the object, and the four predictors comprised the place and time retrieval accuracies (irrespective of confidence) and the interaction between accuracy and confidence for each of the two sources. The factor "subject" was included as a random effect in the model. The logistic regression was implemented in Matlab R2017a. See also Supplementary Material for additional tests concerning the behavioral performance both for the episode retrieval phase and for the source discriminations.

#### Univariate intra-regional fMRI analyses

The univariate intra-regional analysis was based on a random effects approach^82^, and comprised two steps: first-level analyses fitting the BOLD time-series for each participant followed by the second-level analyses for statistical inference at the group level, using SPM12.

The first-level general linear models (GLMs) for the univariate analyses comprised 10 conditions. Five predictors modeled the episode retrieval phase of the trial, four predictors modeled the source discriminations, and one predictor included all confidence judgments. The episode retrieval phase was categorized as a function of the old/new status of the probe and the participants' responses. Accordingly, the 5 conditions included: old trials with "Rem" responses (***Rem***), old trials with "Fam" responses (***Fam***), old trials incorrectly judged as "New" (***ErrOld***), new trials correctly judged as "New" (***New***), and new trials incorrectly judged as "Rem" or "Fam" (***ErrNew***), see also Supplementary Table S1 reporting the average number of trials for each condition. Each event was modeled using the canonical Hemodynamic Response Function in SPM12, time-locked to the presentation of the episode-retrieval display, with a duration of 2.5 s that corresponds to the mean response time for this phase of the trial.

The 4 source discrimination predictors crossed the factors "type of source" (place/time) and "accuracy" of the discrimination (correct / wrong), yielding to the 4 conditions: *PlaceCorr*, *PlaceErr*, *TimeCorr* and *TimeErr*. Each event was time-locked to the presentation of the source-discrimination display with a duration of 3 s (i.e. the mean response time for this phase of the trial). Finally, all confidence judgments were modeled using a single predictor, irrespective of the source (place/time) and response (high/low). The events were time-locked to the confidence judgment display, with a duration of 1.2 s (the mean response time). The six parameters of head movements resulting from the rigid-body realignment were included as covariates of no interest. The time series at each voxel were high-pass filtered at 128 s and pre-whitened by means of autoregressive model AR(1).

The source-discrimination and confidence-judgment predictors were included in the first-level GLMs to account the corresponding variance in the BOLD time-series, but the group analyses (i.e., univariate intra-regional activation and effective connectivity) specifically targeted the episode-retrieval phase of the trial. Accordingly, for each participant, 5 contrasts averaged the parameter estimates across the two fMRI-runs, separately for the 5 episode-retrieval conditions. An additional contrast took the average of the 5 conditions, and of the two runs, with the aim of indentifying regions responding to all episode-retrieval conditions (see "Analyses of effective connectivity", below).

The second-level group analysis testing for changes of intra-regional activation comprised a within-subject ANOVA with the 5 episode-retrieval conditions (with non-sphericity correction^83^). In this ANOVA, we carried our main contrast that compared *Rem* vs *Fam* conditions, testing for voxels with greater activity when the participants correctly recognized an old object and subjectively felt that they could remember additional place/time information about the episode (*Rem*), as compared with correct object-recognition but without any spatio-temporal details about the episode (*Fam*). The ANOVA also allowed us to test the effect of correct "old vs. new" judgments ("*Rem* + *Fam*" vs. "*New*") and the interaction between memory and accuracy ("*Rem* + *Fam* vs. *ErrOld*" vs. "*New* vs. *ErrNew*"). A separate one sample t-test made use of the additional contrast (average of the 5 conditions, cf. above) in order to identify the input region for the analyses of connectivity (FusG: left fusiform gyrus, see below). All statistical thresholds were set to p-FWE = 0.05 corrected for multiple comparisons at voxel-level considering the whole brain as the search volume (corresponding to a T-value = 4.9) and a minimum cluster-size threshold of 10 voxels.

#### Effective connectivity (DCM)

The aim of the current study was to test the hypothesis that the retrieval of episodes encoded in a rich spatio-temporal context relies on the dynamic interplay between regions belonging to the PM memory system^37^. In particular, we expected that the precuneus (PreC) would interact with the medial temporal cortex when the retrieval entails the subjective sense of recollecting not just the memorized-objects but also elements of the encoding context (here place and time, see Introduction). On the basis of the results of the univariate analyses (cf. Fig. 3), we used Dynamic Causal Modeling^49^, to investigate the pattern of inter-regional connectivity explaining the activation of the Precuneus (PreC), the hippocampus/parahippocampus (HC/PHC) and the ventro-medial prefrontal cortex (vmPFC) during successful remembering (i.e., correct retrievals with *Rem-*responses). Specifically, we compared models of effective connectivity where *Rem*-responses did—or did not—modulate the connectivity between PreC and HC/PHC (Family 1 vs. 2, see below); as well as models that did—or did not—comprise modulation of the direct connections between the network's input region (i.e., fusiform gyrus, FusG) and the PreC/vmPFC, and of the connection between HC/PHC and vmPFC, see "model space" below.

##### GLM and ROIs

For the DCM analysis we constructed a new set of single-subject GLMs. The 2 fMRI-runs were concatenated and the GLM now included 7 predictors. Three predictors served to account for the modulatory effect of the object's memory status during correct episode retrievals (see B-parameters of the DCMs, below) and comprised the 3 main conditions of interest: old-objects with *Rem* responses, old-objects with *Fam* responses and new-objects with *New* responses. The remaining four predictors coded for the different types of external input driving the model (C-parameters): one predictor modeled all episode-retrieval events (irrespective of old/new objects and the participant response), one modeled all "Place" source-discrimination events (irrespective of accuracy), one all "Time" source-discrimination events (irrespective of accuracy) and one all confidence-judgment events (irrespective of high/low response). The events' time-locking and durations were the same as in the GLMs used for the univariate analyses. Additional regressors of no interest modeled the main effect of fMRI-run and the realignment parameters. The time series were high-pass filtered at 128 s and pre-whitened by means of autoregressive model AR(1).

Following the estimation of the GLM, for each participant, we extracted the eigenvariate of 4 ROIs: the PreC, the left HC/PHC, the vmPFC and the left fusiform gyrus (FusG). A combination of anatomical and functional constraints served to identify the participant-specific ROIs. Each ROI consisted of a sphere (8 mm radius) centered at the maximum of the omnibus F-contrast of the individual GLM, within a search volume that was defined at the group level. For the PreC, left HC/PHC and vmPFC the search volumes comprised voxels that activated in the contrast "*Rem vs Fam*" in the univariate analysis (see Fig. 3). The search was further constrained using the maximum probability maps of the Harvard–Oxford atlas (https://neurovault.org/collections/262/), considering the PreC, the left hippocampus and vmPFC. It should be noted that because these additional anatomical constraints were used only to define the center of the spherical ROIs, not all the voxels in each individual ROI belonged to the selected anatomical region. In particular, the ROIs in the medial temporal cortex did include also voxels in the parahippocampus (cf., also activation cluster for the main group analysis). Considering all the voxels in each HC/PHC ROIs, out of the 19 participants, 16 had more voxels in HC than in PHC vs. 3 participants with more voxels in PHC than HC. On average, across subjects, 68.5% (std = 18.5) of the voxels had higher probability to be in HC than PHC. Hence, also for the DCM analysis both the HC and the PHC contributed to the results, albeit with a prevalence of signal arising from the hippocampus. The search volume for individual maxima located in the left FusG comprised voxels showing mean activation for all 5 episode-retrieval conditions (group-level one sample t-test, cf. "univariate analyses", above) and that belonged to the fusiform gyrus according to the Harvard–Oxford atlas. In order to obtain search regions of analogous size for the 4 search volumes, the identification of the HC/PHC and vmPFC voxels in the group analysis was done a p-unc. = 0.001, rather than p-FWE-corr = 0.05. With this, the number of voxels in each search region was: PreC (183), HC/PHC (113), vmPFC (199) and FusG (186). The localization of the final subject-specific ROIs is shown in Fig. 4A.

##### Model space

In order to test possible mechanisms of how episodic recollection affects the inter-regional dynamics between regions of the medial memory system, we constructed a series of DCMs and used Bayesian model selection to identify the model that best explained our fMRI data.

All models included the 4 ROIs and driving input entering the network at the level of the left FusG. The driving input (C-parameters) comprised all phases of the retrieval trial: that is, the episode retrieval, place and time source-discriminations and the confidence judgments (cf. corresponding predictors in the GLM, above). The intrinsic connectivity of the models (A-parameters) were also fixed and included bidirectional connections between FusG and the three regions of the medial memory systems; plus bidirectional connections between the HC/PHC and PreC, and between HC/PHC and vmPFC; see also Fig. S2.

Eight models differed with regards to how episode retrievals with *Rem*-responses modulated the connectivity between the nodes of the network (B-parameters). To test the main hypothesis about the role of the PreC-HC/PHC connectivity, the models were partitioned in two families^50^. Family 1 comprised four models with modulation of the HC/PHC-PreC connection, while family 2 included four models without any modulation of the HC/PHC-PreC connection. Within each family, the different models comprised—or not—the modulation of the direct connections between the input region (FusG) and the PreC/vmPFC, and of the HC/PHC-vmPFC connection. By doing this we could effectively test the role of the medial temporal cortex that—in the models with the direct connections switched off—was the only path permitting the *Rem* condition to influence the activation in PreC and/or vmPFC, see also Supplementary Fig. S2 with the 8 models that defined the whole DCM model space.

##### Model selection and parameter inference

The DCM analyses were carried out using SPM12 (https://www.fil.ion.ucl.ac.uk/spm/software/spm12/). Fixed-effects Bayesian model selection (BMS-FFX) was conducted to identify the most probable family and the most probable model. The fixed-effects model selection relies on the computation of group Bayes factors that sum the model log-evidences across all participants, providing us with the relative probability that the data were generated by one model relative to another^52^. We then used fixed-effect Bayesian Parameter Averaging^54^. (BPA) on the winning model to test the significance of the modulatory B-parameters. We report modulations with posterior probabilities > 99%.

### Supplementary Information

Supplementary Information.

## Data Availability

The datasets generated during and analysed during the current study are available from the corresponding author on reasonable request.
