# Supplementary material for: The influence of the precuneus on the medial temporal cortex determines the subjective quality of memory during the retrieval of naturalistic episodes
Source: Sci Rep. 2024 Apr 4;14:7943. doi: 10.1038/s41598-024-58298-y (PMC10995201; doi:10.1038/s41598-024-58298-y)
Supplement: Supplementary file 1 — Supplementary Information. [file 41598_2024_58298_MOESM1_ESM.docx]

Supplementary Material

**The influence of the precuneus on the medial temporal cortex determines the subjective quality of memory during the retrieval of naturalistic episodes**

*Samy-Adrien Foudil & Emiliano Macaluso*

**1. Supplementary results: Behavior**

1.1. Episode retrieval

The Table S1 (top) reports the average number of trials (+/- std) for the five main conditions associated with the episode-retrieval phase (cf. also fMRI analyses).

In addition, for this phase of the trial we also considered the overall object recognition performance, irrespective of subjective quality of the retrieval (pooling *Rem*/*Fam* responses). The object recognition hit rate was 80.2% [std = 10.4, Hits = (Rem_seen_ + Fam_seen_) / (Rem_seen_ + Fam_seen_ + New_seen_)] and the false alarm rate was 30,3% [std = 12.7, FA = (Rem_unseen_ + Fam_unseen_) / (Rem_unseen_ + Fam_unseen_ + New_unseen_)]. The correct rejections rate was 69.7% (i.e. CR = 1-FA). The corresponding d-prime was 1.51 [std = 0.49], indicating that the participants could perform the object recognition task.

The high Hits rate this was primarily due to trails with *Rem*-responses [Hits_Rem_ = Rem_seen_ / (Rem_seen_ + Rem_unseen_) = 84.4%, std = 8.7, p < 0.001], while performance in trials with *Fam*-responses was not above chance level [Hits_Fam_ = Fam_seen_ / (Fam_seen_ + Fam_unseen_) = 53.3%, std = 16.4, p > 0.3]. Please note that the computation of the Hits rates separately for *Rem* and *Fam* conditions had to use False Alarms rather than Misses in the denominator.

| **EPISODE RETRIEVAL PHASE** | | | | | | | | | | |
| --- | --- | --- | --- | --- | --- | --- | --- | --- | --- | --- |
|  | **seen/old** | | | | | |  | **unseen/new** | | |
|  | *Rem_seen_* | *Fam_seen_* | | *Wrong: New_seen_* | | |  | *Correct: New_unseen_* | | *Wrong (*)* |
|  | 35.6, [10.9] | 12.3 [7.8] | | 12.1 [6.4] | | |  | 41.9 [7.4] | | 18.1 [7.4] |
| **SOURCE DISCRIMINATIONS** | | | | | | | | | | |
|  | **Place** | | | |  | **Time** | | | | |
|  | *High* | | *Low* | |  | *High* | | | *Low* | |
|  | 14.7 [5.8] | | 29.7 [8.3] | | | 24.2 [7.1] | | | 22.8 [8.1] | |

**Supplementary Table S1.** Mean number of trials [+/- std] for the different conditions associated with the episode retrieval phase (top) and the source discrimination task (bottom). (*) Separately for *Rem* and *Fam* trials, the number of false alarms was: Rem_unseen_ 6.8 [std = 4.2], Fam_unseen_ 10.9 [std = 6.8].

1.2. Source discrimination

The average number of trials (+/- std) for the Place and Time source memory discriminations are reported in Table S1 (bottom), separately according to the confidence judgments (cf. also Fig. 2A).

In addition, we examined the accuracy of the source-discriminations as a function of the response that the participants gave in the initial episode-retrieval phase of the trial (*Rem* vs. *Fam*), see Table S2 below. These additional tests confirmed that none of the low-confidence conditions was associated with accuracies above chance level (see bottom part of Table S2). For the high-confidence conditions, accuracy was above chance when the participants responded *Rem* in the episode retrieval phase (Place: p < 0.035, Time: p < 0.001, one-tailed t-tests), and a statistical trend was also found following *Fam* responses in the Time task (p < 0.057, one-tailed, marked with #-symbol in Table S2). Overall, these results align with the logistic regression analyses that also highlighted a relationship between *Rem* responses and source memory, specifically when the participants were confident about their responses (see Fig. 2B).

Nonetheless, it should be noticed that the number of trials that contributed to some of these additional tests was very low and the results should be interpreted with caution (cf. "Num. trials", in Table S2). This was particularly true for high-confident source-discrimination trials following *Fam* responses. Both for the Place and Time tasks, these trials were rare (2-3 on average per subject, and several participants did not have any such response combinations at all: 6 participants for the Place-discrimination and 4 participants for the Time-discrimination).

|  |  | **Rem** |  |  | **Fam** |  |
| --- | --- | --- | --- | --- | --- | --- |
|  |  | Accuracy (%) | Num. trials |  | Accuracy (%) | Num. trials |
| **High confidence** |  |  |  |  |  |  |
| Place |  | **55.7 *** | 12.6 |  | 52.6 | 2.2 |
| Time |  | **65.7 *** | 20.8 |  | **63.6 ^#^** | 3.3 |
| **Low confidence** |  |  |  |  |  |  |
| Place |  | 49.7 | 20.1 |  | 53.0 | 9.7 |
| Time |  | 48.3 | 14.2 |  | 51.3 | 8.6 |

**Supplementary Table S2.** Accuracy and mean number of trials for the source-discrimination tasks, as a function of the subjective quality of the initial episode retrieval response (*Rem*/*Fam*). Highlighted in bold : one tailed t-tests showing source-discrimination accuracies that were larger than the 50% chance level, (*) p < 0.05, (#) p < 0.1.

**2. Supplementary results: Functional imaging**

2.1. Intra-regional activation analyses


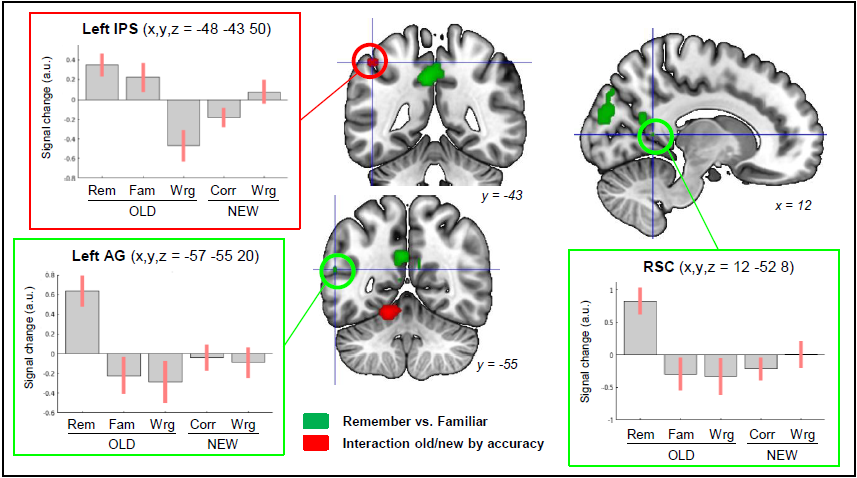


**Supplementary Figure S1. Remembered objects and the effect of correct retrieval irrespective of subjective evaluation.** Intra-regional activations associated with *Rem* responses (vs. *Fam*, rendered in green) here displayed without any minimum-cluster size threshold. Within the PM system, this revealed the activation of two additional small clusters of 3 voxels in the left angular gurus (AG) and the retrosplenial cortex (RSC), see also Table 1. These activations are rendered together with the contrast testing for the effect of correct recognition of see/old objects, irrespective of *Rem/Fam* subjective evaluation (i.e. the interaction old/new by accuracy, activations rendered in red; see also Table 1). The signal plots show the pattern of activation for the AG and the RSC with a selective activation for the *Rem* condition (see bar 1), as well as the activation of the left anterior intraparietal sulcus (IPS) that instead activated both for *Rem* and *Fam* conditions (see bars 1 and 2 of the signal plot). Activations are displayed at p-FWE-corr = 0.05, corrected for multiple comparisons at the whole brain level (T-value = 4.9, min. cluster size = 0 voxels). The signal plots show the parameter estimates at the peak-voxel and for all the episode-retrieval conditions. These include correctly recognized new-items (“NEW Corr”) and incorrect responses (“Wrg”, plotted separately for old/seen and new/unseen trials). The parameter estimates of the general linear model are mean-adjusted (sum = 0) and negative values should not be interpreted as de-activations. The images were made with Statistical Parametric Mapping software SPM12 (Wellcome Department of Imaging Neuroscience, University College London, UK; http://www.fil.ion.ucl.ac.uk/spm).

2.2. Inter-regional effective connectivity (DCM)

**
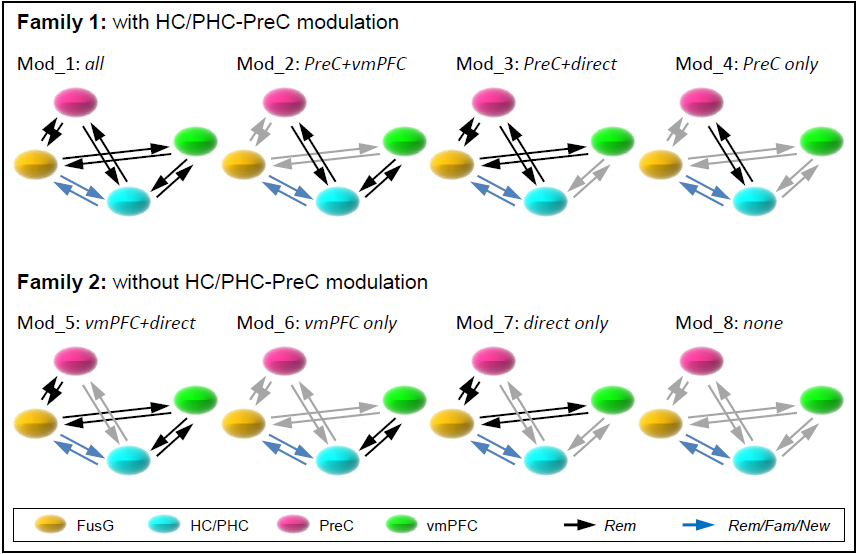
**

**Supplementary Figure S2. DCM model space.** The model space included 8 models, partitioned in two families. All models received external input at the FusG node (C-parameters, not shown; see Methods for details). The intrinsic connectivity (A-parameters) was the same in all models and it is represented by the arrows, irrespective of color. The models differed in terms of the modulatory influence that the *Rem* condition could exert on specific connections (B-parameters, black arrows). These modulations entailed the connectivity between HC/PHC-PreC, HC/PHC-vmPFC and the direct connections between FusG and PreC/vmPFC. The two families included the models with/without modulation of the HC/PHC-PreC connections. In addition, in all models the three retrieval conditions (*Rem/Fam/New*) could modulate the FusG-HC/PHC connections (blue arrows). FusG: left fusiform gyrus; HC/PHC: left hippocampus/parahippocampus, PreC: precuneus, vmPFC: ventro-medial prefrontal cortex. *Rem/Fam*: correctly recognized old-objects with “Remember/Familiar” responses, *New*: correctly recognized new-objects.

**
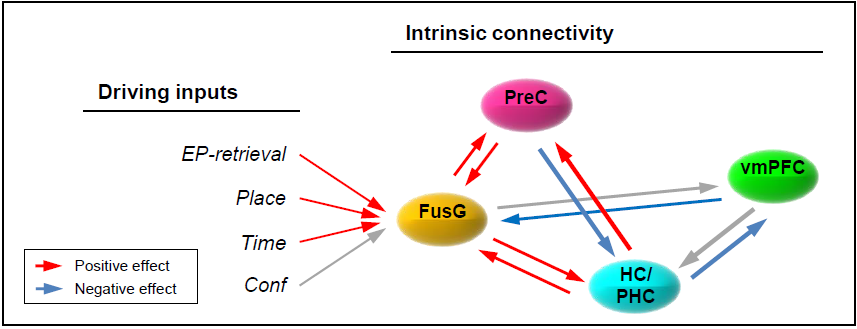
**

**Supplementary Figure S3. Intrinsic connectivity and driving inputs for the winning model.** Bayesian Parameter Averaging showed significant couplings between the different regions of the network (A-parameters of the DCM, see also Methods section). The condition-independent connectivity between the precuneus and the hippocampus/parahippocampus indicated a “driving” effect of the HC/PHC to the PreC, while the PreC connection exhibited a negative influence on the HC/PHC. This pattern was opposite to the modulatory effect specific for the “Rem” responses (cf. Fig. 4A, and note that the HC/PHC-vmPFC connectivity showed a related pattern). Concerning the driving input (C-parameters), the presentation of the object-image during the episode retrieval phase of the trial, as well as the two source discrimination tasks elicited significant activation of the network at the level of the fusiform gurus, while the presentation of the confidence judgments did not. FusG: left fusiform gyrus; HC/PHC: left hippocampus/parahippocampus, PreC: precuneus, vmPFC: ventro-medial prefrontal cortex. *EP-retrieval*: the episode retrieval phase of the trial, *Place*: place-discrimination phase, *Time*: Time-discrimination phase, Conf: confidence judgment (see also Fig. 1B). Red/blue arrows: posterior probability > 99%.

**
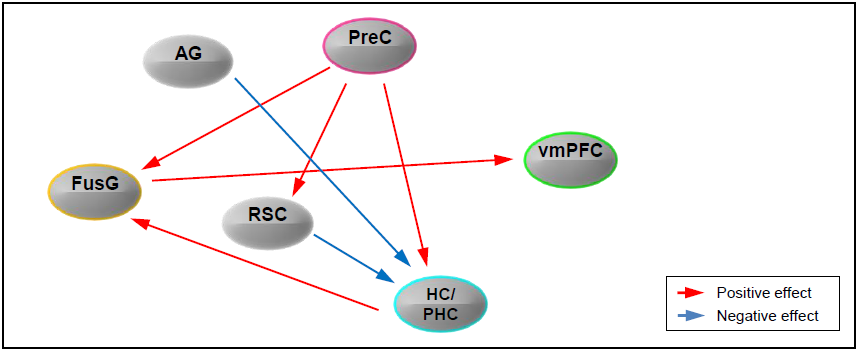
**

**Supplementary Figure S4. Exploratory analysis using Parametric Empirical Bayes and Bayesian Model Reduction.** Results of an additional analysis of effective connectivity that included the left angular gyrus (AG) and the retrosplenial cortex (RSC), as well as the precuneus (PreC), the left hippocampus/ parahippocampus (HC/PHC), the ventro-medial prefrontal cortex (vmPFC), and the fusiform gyrus (FusG). The analysis considered a fully connected model, where the subjective quality of retrieval (*Rem*-responses) could modulate all the connections. An iterative procedure removed any parameter that did not contribute to the model evidence and Bayesian model averaging was used to average the models from the last iteration. This resulted in the identification of 7 parameters with a posterior probability > 99% (red/blue arrows in the figure). Both the AG and the RSC were found to have a negative effect of the HC/PHC, while the PreC exerted a positive influence on the HC/PHC (as in the main DCM analysis, cf. Fig. 4). It is important to stress that the results of this exploratory analysis should not be assessed against the main DCM results, because different data were used for the two analyses.
